# Time-Varying Effects of Parental Alcoholism on Depression

**DOI:** 10.5888/pcd14.170100

**Published:** 2017-12-14

**Authors:** Sunita Thapa, Arielle S. Selya, Yvonne Jonk

**Affiliations:** 1 Department of Health Policy, Vanderbilt University School of Medicine, Nashville, Tennessee; 2 Master of Public Health Program, Department of Population Health, University of North Dakota, Grand Forks, North Dakota

## Abstract

**Introduction:**

Children of alcoholic parents are at increased risk for lifetime depression. However, little is known about how this risk may change in magnitude across age, especially in mid-adulthood and beyond.

**Methods:**

We used a nationally representative sample (N = 36,057) of US adults from the National Epidemiologic Survey on Alcohol and Related Conditions, wave III. After adjusting for demographic characteristics, we examined the relationship between parental alcoholism and outcomes of 1) major depressive disorder, Diagnostic and Statistical Manual of Mental Disorders-5th edition (DSM-5) and 2) DSM-5 persistent depressive disorder. To examine continuous moderation of this relationship across participants’ age, we used time-varying effect models.

**Results:**

Parental alcoholism was associated in general with a higher risk for both major depressive disorder (odds ratio [OR], 1.98, 95% confidence interval [CI], 1.85–2.11; *P* < .001) and persistent depressive disorder (OR, 2.28, 95% CI, 2.04–2.55; *P* < .001). The association between parental alcoholism and major depressive disorder was stable and positive across age, but the association with persistent depressive disorder significantly declined among older adults; respondents older than 73 years old were not at increased risk for persistent depressive disorder.

**Conclusions:**

Findings from this study show that the risk of parental alcoholism on depression is significant and stable among individuals of a wide age range, with the exception of a decline in persistent depressive risk among older adults. These findings highlight the importance of screening for depression among adults with parental alcoholism.

**MEDSCAPE CME**

Medscape, LLC, is pleased to provide online continuing medical education (CME) for this journal article, allowing clinicians the opportunity to earn CME credit.

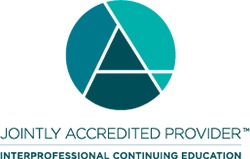
In support of improving patient care, this activity has been planned and implemented by Medscape, LLC, and *Preventing Chronic Disease*. Medscape, LLC, is jointly accredited by the Accreditation Council for Continuing Medical Education (ACCME), the Accreditation Council for Pharmacy Education (ACPE), and the American Nurses Credentialing Center (ANCC), to provide continuing education for the healthcare team.

Medscape, LLC, designates this Journal-based CME activity for a maximum of 1.00 *AMA PRA Category 1 Credit(s)™*. Physicians should claim only the credit commensurate with the extent of their participation in the activity.

All other clinicians completing this activity will be issued a certificate of participation. To participate in this journal CME activity: (1) review the learning objectives and author disclosures; (2) study the education content; (3) take the post-test with a 75% minimum passing score and complete the evaluation at http://www.medscape.org/journal/pcd; (4) view/print certificate.

**Release date: December 14, 2017; Expiration date: December 14, 2018**

**Learning Objectives**

Upon completion of this activity, participants will be able to:

1. Evaluate risk for lifetime major depressive disorder among children of alcoholic parents, based on a national database study using the National Epidemiological Survey on Alcohol and Related Conditions, wave III
2. Determine risk for lifetime persistent depressive disorder among children of alcoholic parents, based on a national database study using the National Epidemiological Survey on Alcohol and Related Conditions, wave III
3. Assess clinical implications regarding risk for lifetime depression among children of alcoholic parents, based on a national database study using the National Epidemiological Survey on Alcohol and Related Conditions, wave III

**EDITOR**

Camille Martin, RD, LD

Editor, *Preventing Chronic Disease*

Disclosure: Camille Martin, RD, LD, has disclosed no relevant financial relationships.

 

**CME AUTHOR**

Sunita Thapa, MPH

Department of Health Policy, Vanderbilt University School of Medicine, Nashville, Tennessee

Disclosure: Sunita Thapa, MPH, has disclosed no relevant financial relationships.

 

**AUTHORS**

Arielle S. Selya, PhD

Master of Public Health Program, Department of Population Health, University of North Dakota, Grand Forks, North Dakota

Disclosure: Arielle S. Selya, PhD, has disclosed the following relevant financial relationships:

Received grants for clinical research from: Sanford Research

Yvonne Jonk, PhD

Master of Public Health Program, Department of Population Health, University of North Dakota, Grand Forks, North Dakota

Disclosure: Yvonne Jonk, PhD, has disclosed no relevant financial relationships.

## Introduction

Parental alcoholism has various negative physical, mental, and social consequences. Chief among these is depression; offspring of alcoholics are at heightened risk of depressive mood symptoms (1,2). The evidence for heightened depression among those exposed to parental alcoholism is particularly strong among young, college-aged adults (3,4).

Much of the research on the association between parental alcoholism and depression focuses on the question of resilience among adult children of alcoholics; that is, whether these individuals are ever able to overcome the challenges of parental alcoholism. Although some evidence suggests that older adults (those in their late 20s and early 30s) are more resilient than are young adults (those aged 18 through their early 20s) (5), there is little research on the effects of parental alcoholism among offspring of alcoholics in mid- to late adulthood, making their longer-term resilience unknown. Furthermore, the question of increased resilience at older ages assumes that the magnitude of the effect of parental alcoholism changes with increasing age; however, such age-varying effects have not yet been examined.

This study examined 1) the association between parental alcoholism and lifetime outcomes of both major depressive disorder (MDD) and persistent depressive disorder (PDD) among a full range of adults after controlling for demographic characteristics and 2) the age-varying effects of these associations (ie, how they may change in strength across participants’ ages). We used data from wave III of the National Epidemiologic Survey on Alcohol and Related Conditions (NESARC-III), a large nationally representative data set.

### Methods

NESARC-III was sponsored, designed, and directed by the National Institute on Alcohol Abuse and Alcoholism (NIAAA) and conducted during 2012–2013. NESARC-III is a nationally representative sample of the civilian noninstitutionalized population of the United States aged 18 years or older; it had a 61.1% response rate and an original sample size of 36,309. The NIAAA collected information via questionnaires on alcohol and drug use and disorders, related risk factors, and associated physical and mental disabilities on the basis of NIAAA’s Alcohol Use Disorder and Associated Disabilities Interview Schedule. This study excluded respondents with missing information on parental alcoholism; the final sample size for this study was 36,057. We used existing data from human participants in NESARC, and the study was approved by the University of North Dakota institutional review board. We completed the final analyses in May of 2016.

### Measures

#### Parental alcoholism

Parental alcoholism was based on the self-reported answer to the question “Before you were 18, parent/other adult living in home was a problem drinker/alcoholic?” as a binary response variable (yes or no).

#### Depression

We analyzed 2 depressive disorders, lifetime MDD and lifetime PDD, as separate outcomes. Each outcome was derived from detailed self-reported responses to questionnaire items on the basis of corresponding criteria from the Diagnostic and Statistical Manual of Mental Disorders, 5th edition (DSM-5)(6). Briefly, lifetime MDD is characterized by one or more discrete episodes of at least 2 weeks during which respondents had either a depressed mood or a loss of interest in nearly all activities at some time during their adult lives (6). Lifetime PDD is a milder but more chronic form of depression and can be diagnosed when the mood disturbance continues for at least 2 years at some time during an adult’s life (6). Both MDD and PDD exclude mood or anxiety disorders that are either substance-induced or due to a general medical condition.

#### Demographic characteristics

Age and sex were self-reported. Race/ethnicity was self-reported as white, black, Hispanic, American Indian, or Asian. Full-time employment was self-reported as working 35 or more hours per week or less than 35 hours per week.

Marital status was self-reported according to 6 response options, which were re-categorized as currently married (ie, married or living with someone as if married), not currently married (ie, widowed, divorced, or separated), and never married.

Education was self-reported with 14 response levels ranging from “no formal schooling” to “completed Master’s degree or higher,” and we re-categorized these into 3 levels: less than a high school diploma, high school diploma, and some college or more.

Annual household income was self-reported with 21 response categories ranging from less than $5,000 to $200,000 or more. We recoded these into a new numeric variable on the basis of midpoints of each category up to level 20; level 21 (≥$200,000) was recoded as $250,000, which is approximately the median income among households earning $200,000 or more (7).

### Statistical analyses

We conducted weighted regressions using the statistical software R (The R Foundation) and its survey package to examine the association between parental alcoholism and outcomes of MDD and PDD, after adjusting for demographic characteristics.

We used time-varying effect models (TVEMs), an extension of regression modeling that allows coefficients to vary continuously over time (8), to assess how the association between parental alcoholism and depression outcomes varied across age of participants. In other words, TVEMs examine moderation across some continuous measure of time (eg, historical time, age, time from event). TVEMs are spline-based regression models, which estimate a lower-order polynomial trend within equal intervals on the basis of user-specified number of knots, *k*. On the basis of established standards for this methodology (9), 10 knots were specified, and P-spline estimation, which automatically finds the most parsimonious model (*k* ≤10), was used. We ran separate logistic TVEM models for outcomes of MDD and PDD after controlling for demographic characteristics. Each model included a time-varying intercept (to adjust for the overall prevalence of depression across age) and the time-varying predictor of age (to examine continuous moderation of the effect of parental alcoholism across ages). We performed TVEM analyses in SAS 9.3 (SAS Institute Inc) using a publicly available SAS macro (9), version 3.1.0. TVEM analyses were interpreted with respect to 1) overall significance of the effect at a given value of age (ie, whether the confidence bands overlap the odds ratio (OR) of 1.0), and 2) the change in the effect across different ages (ie, whether the confidence bands exclude each other at different ages). Although these methods of establishing significance are more conservative than conventional significance tests, we did this because *P* values were available only for time-invariant covariates.

## Results

Approximately 23% of respondents (n = 8,407) reported parental alcoholism. Respondents who reported parental alcoholism were significantly more likely than adults who did not report parental alcoholism to meet DSM-5 criteria for both MDD (29.6% vs 17.7%, *P* < .001) and PDD (9.3% vs 4.4%, *P* < .001) (Table). People who reported parental alcoholism were slightly but significantly younger (mean age, 44.8 y vs 45.9 y, *P* < .001); were more likely to be female (59.4% vs 55.4%, *P* < .001); had lower annual household incomes (median $32,500 vs $37,500, *P* < .001); were less likely to be never married (25.8% vs 28.4%, *P* < .001); were more likely to be not currently married (27.6% vs 25.4%, *P* < .001); were more likely to be white (57.8% vs 51.4%) or American Indian (2.1% vs 1.2%); and were less likely to be black (18.2% vs 22.3%) or Asian (1.9% vs 5.9%). The 2 groups did not significantly differ by education level (approximately 15% had <high school diploma, 22% high school diploma, and 62% some college or more), or full-time employment status (approximately 43%).

**Table:** Descriptive Statistics of Sample (N = 36,057), Study on Effects of Parental Alcoholism on Depression, National Epidemiological Survey on Alcohol and Related Conditions, Wave III, 2012–2013

Measure	Parental Alcoholism^a^	
	Yes	No
**Major depressive disorder^b^**	29.6	17.7
**Persistent depressive disorder^b^**	9.3	4.4
**Median (IQR), age, y^c^**	44.0 (32–56)	44.0 (30–59)
**Sex^b^**		
Female	59.4	55.4
Male	40.6	44.6
**Education**		
<High school diploma	15.7	14.8
High school diploma	22.4	22.7
Some college or more	61.9	62.4
**Median (IQR) annual household income, $^c^**	32,500 (17,500–65,000)	37,500 (17,500–65,000)
**Full-time employment (≥35 h/wk)**	43.2	44.2
**Marital status**		
Currently married	46.6	46.2
Not currently married^b^	27.6	25.4
Never married^b^	25.8	28.4
**Race/ethnicity**		
White^b^	57.8	51.4
Black^b^	18.2	22.3
American Indian^b^	2.1	1.2
Asian^b^	1.9	5.9
Hispanic	19.9	19.2

Abbreviation: IQR, interquartile range.

a Numeric variables presented as median (IQR), and categorical variables presented as percentages.

b χ^2^ significant in parental alcoholism status at *P* < .05. MDD is characterized by discrete episodes of at least 2 weeks during which respondents experienced either depressed mood or a loss of interest in nearly all activities in adults at some time in their lives. Lifetime PDD is a milder but more chronic form of depression and can be diagnosed when the mood disturbance continues for at least 2 years in adults at some time in their lives (6).

c Analysis of variance significant in parental alcoholism status at *P* < .05.

Additionally, compared with respondents who did not report parental alcoholism, those who reported parental alcoholism were slightly but significantly younger when they first had the first episode of MDD (median age, 27.8 y vs 30.5 y, *P* < .001) and PDD (median age, 27.9 y vs 30.6 y, *P* < .001) and had a significantly higher number of MDD episodes (median no., 4.6 vs 3.5, *P* < .001) and a nonsignificantly higher number of PDD episodes (median no., 2.1 vs 1.9). Respondents who reported parental alcoholism also talked to any health professional or therapist significantly more often to help improve their mood caused by MDD (63% vs 58%, *P* < .001) and nonsignificantly more often to help improve their mood caused by PDD (68% vs 64%) compared with respondents who did not report parental alcoholism. Respondents who reported parental alcoholism were significantly more likely to have symptoms of suicidal ideation (13% vs 8%, *P* < .001) and also meet DSM-5 criteria for other mental comorbidities such as anxiety (21% vs 11%, *P* < .001), personality disorders (27% vs 12%, *P* < .001), eating disorders (3% vs 1.5%, *P* < .001), substance use disorders (57% vs 37%, *P* < .001), and posttraumatic stress (12% vs 5%, *P* < .001).

Weighted regression analyses showed that parental alcoholism was associated with an approximately twofold increase in the odds of both MDD (OR, 1.84; 95% confidence interval [CI], 1.72–1.96; *P* < .001) and PDD (OR, 2.11; 95% CI, 1.88–2.37; *P* < .001), after controlling for demographics.

**Figure 1:**
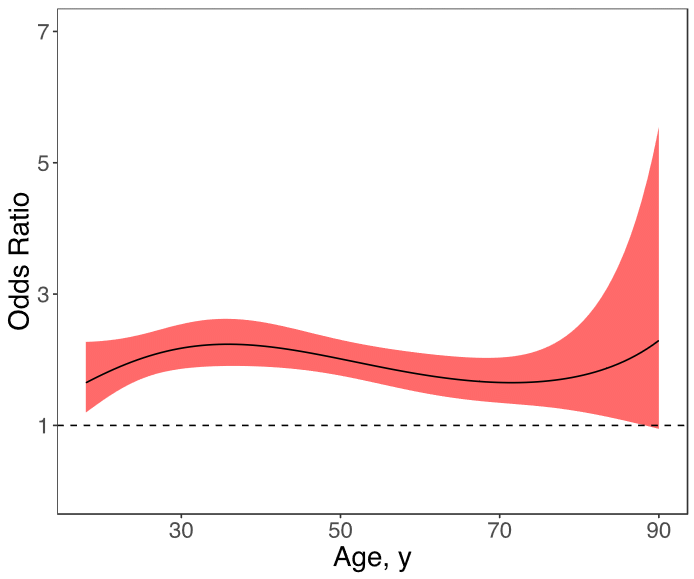
Age-varying effects of parental alcoholism on lifetime major depressive disorder for respondents aged 18–90 years, National Epidemiologic Survey on Alcohol and Related Conditions, Wave III, 2012–2013. Age-varying effects are presented as odds ratios (ORs) across ages; the solid line represents the OR point estimates, and the surrounding shading represents 95% confidence intervals. The horizontal line represents an OR of 1.00. **Table:** 

Parental alcoholism had a positive and stable effect on MDD across individuals throughout most of the age range of respondents aged 18 to 85 years (Figure 1). Participants between these ages were approximately 2 times as likely to have MDD as were participants who reported no parental alcoholism. Because of the small sample size of participants older than 85 years and the resulting widening of the confidence band (ie, the lower limit of the confidence band is less than the OR of 1), the relationship was no longer significant among these individuals, even though the point estimate remained stable.

Age, y	Parental Alcoholism Lower Limit	Parental Alcoholism Upper Limit	Parental Alcoholism Estimate (Odds Ratio)
18.0	1.195427179	2.272260309	1.648126734
18.7272727	1.260284189	2.275778416	1.69355471
19.4545455	1.323772899	2.280927531	1.737650728
20.1818182	1.385351709	2.287849951	1.780302458
20.9090909	1.444477368	2.296690058	1.821405175
21.6363636	1.500622699	2.307580984	1.860862275
22.3636364	1.553300166	2.320625326	1.898585711
23.0909091	1.60209056	2.335870551	1.934496358
23.8181818	1.646673787	2.353282051	1.968524287
24.5454545	1.686856114	2.37271941	2.000608968
25.2727273	1.722586815	2.393922902	2.030699393
26.0	1.753958399	2.416515995	2.05875412
26.7272727	1.781188888	2.440025357	2.084741244
27.4545455	1.804590073	2.463914416	2.108638304
28.1818182	1.824529489	2.487622715	2.130432116
28.9090909	1.841394202	2.510602989	2.150118552
29.6363636	1.85556197	2.532350392	2.167702259
30.3636364	1.867381868	2.552421807	2.183196327
31.0909091	1.877163724	2.570445928	2.196621918
31.8181818	1.885174314	2.586126186	2.208007849
32.5454545	1.891638012	2.599238875	2.217390145
33.2727273	1.896739946	2.609628446	2.224811569
34.0	1.900630285	2.617201424	2.230321118
34.7272727	1.903428782	2.621919841	2.23397352
35.4545455	1.905229075	2.623794732	2.235828708
36.1818182	1.906102546	2.622879964	2.235951291
36.9090909	1.906101639	2.619266515	2.234410033
37.6363636	1.905262677	2.613077218	2.231277324
38.3636364	1.903608223	2.604461965	2.226628666
39.0909091	1.901149081	2.593593312	2.220542173
39.8181818	1.897886019	2.580662423	2.213098084
40.5454545	1.893811306	2.565875303	2.204378293
41.2727273	1.888910167	2.549449243	2.194465902
42.0	1.883162224	2.531609402	2.183444799
42.7272727	1.876543032	2.512585464	2.17139926
43.4545455	1.869025779	2.492608297	2.158413575
44.1818182	1.8605832	2.471906558	2.144571709
44.9090909	1.851189775	2.45070322	2.129956981
45.6363636	1.840824171	2.429212002	2.114651785
46.3636364	1.829471908	2.40763378	2.098737326
47.0909091	1.817128103	2.386153072	2.082293401
47.8181818	1.803800135	2.364934788	2.065398192
48.5454545	1.789509965	2.344121462	2.048128101
49.2727273	1.774295891	2.323831223	2.030557606
50.0	1.758213466	2.304156723	2.012759145
50.7272727	1.741335442	2.285165178	1.994803027
51.4545455	1.723750686	2.266899561	1.976757363
52.1818182	1.705562128	2.249380853	1.958688029
52.9090909	1.686883971	2.232611154	1.940658643
53.6363636	1.667838406	2.216577355	1.922730569
54.3636364	1.648552156	2.201255072	1.904962938
55.0909091	1.629153115	2.186612553	1.887412687
55.8181818	1.609767297	2.17261433	1.87013462
56.5454545	1.590516228	2.159224489	1.853181478
57.2727273	1.571514848	2.146409484	1.836604033
58.0	1.552869893	2.134140499	1.820451189
58.7272727	1.534678724	2.122395428	1.804770098
59.4545455	1.517028509	2.111160516	1.789606295
60.1818182	1.499995669	2.100431798	1.775003831
60.9090909	1.4836455	2.090216384	1.761005432
61.6363636	1.468031882	2.08053371	1.74765266
62.3636364	1.453197003	2.07141681	1.734986081
63.0909091	1.439171043	2.062913685	1.723045455
63.8181818	1.42597177	2.055088813	1.711869923
64.5454545	1.413604026	2.048024841	1.701498211
65.2727273	1.402059091	2.041824467	1.69196884
66.0	1.391313947	2.03661252	1.683320351
66.7272727	1.381330494	2.032538193	1.675591533
67.4545455	1.372054796	2.029777369	1.668821672
68.1818182	1.363416488	2.028534933	1.663050803
68.9090909	1.355328512	2.029046931	1.658319981
69.6363636	1.347687402	2.031582382	1.654671563
70.3636364	1.340374316	2.036444577	1.652149511
71.0909091	1.333257058	2.043971681	1.650799706
71.8181818	1.326193189	2.054536565	1.650670288
72.5454545	1.319034267	2.068545902	1.651812013
73.2727273	1.311631011	2.086438791	1.654278641
74.0	1.303839047	2.10868534	1.658127342
74.7272727	1.295524668	2.135785855	1.663419147
75.4545455	1.286570012	2.168271365	1.670219421
76.1818182	1.276877083	2.206706168	1.678598384
76.9090909	1.266370228	2.25169296	1.688631673
77.6363636	1.254996934	2.303880859	1.700400957
78.3636364	1.242727071	2.363976434	1.713994606
79.0909091	1.229550899	2.432757678	1.729508424
79.8181818	1.21547627	2.511090838	1.747046458
80.5454545	1.200525457	2.599950042	1.766721884
81.2727273	1.184731956	2.700439852	1.788657985
82.0	1.168137532	2.813821052	1.812989238
82.7272727	1.150789662	2.941540212	1.839862513
83.4545455	1.132739419	3.085263815	1.869438403
84.1818182	1.11403981	3.246917982	1.901892713
84.9090909	1.094744514	3.42873509	1.937418109
85.6363636	1.07490697	3.633308915	1.976225968
86.3636364	1.054579739	3.86366024	2.01854844
87.0909091	1.03381408	4.123315369	2.064640764
87.8181818	1.012659698	4.416400501	2.114783865
88.5454545	0.991164591	4.747755626	2.16928727
89.2727273	0.969375	5.123072466	2.228492399
90.0	0.947335398	5.549062135	2.292776262

**Figure 2:**
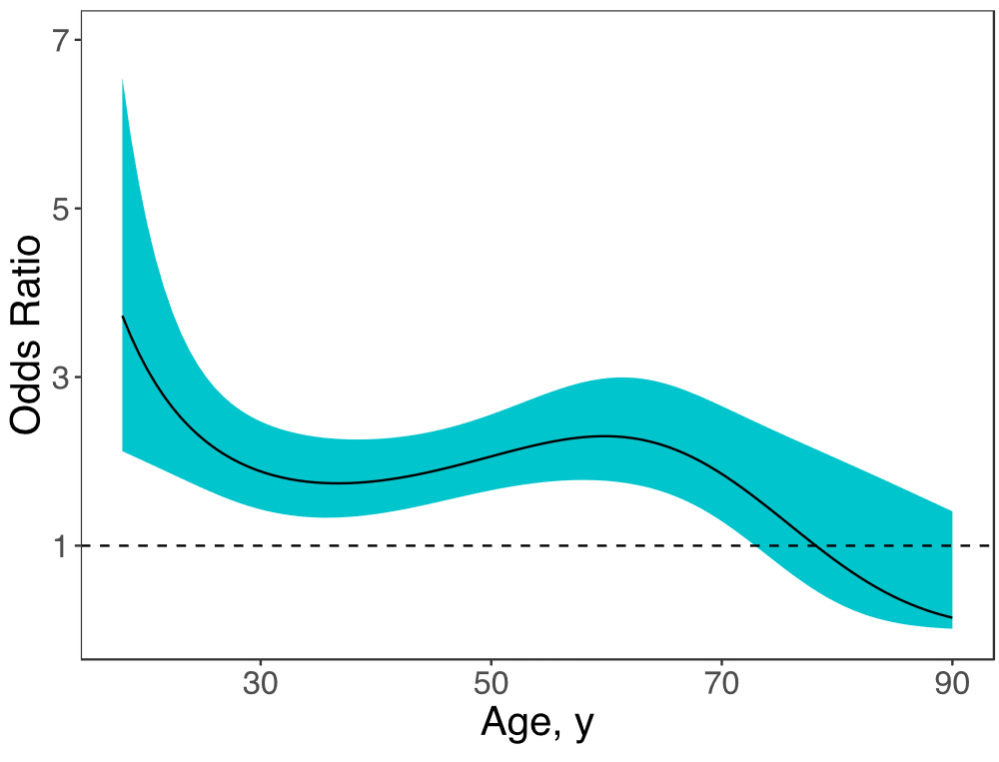
Age-varying effects of parental alcoholism on lifetime persistent depressive disorder for respondents aged 18–90 years, National Epidemiologic Survey on Alcohol and Related Conditions, Wave III, 2012–2013. Age-varying effects are presented as odds ratios (ORs) across ages; the solid line represents the OR point estimates, and the surrounding shading represents 95% confidence intervals. The horizontal line represents an OR of 1.00. **Table:** 

Similarly, parental alcoholism had a positive effect on PDD across a wide age range (Figure 2). Participants aged 18 to 73 years were approximately 2 times as likely to have PDD as were participants who reported no parental alcoholism. The association was nonsignificant for those aged 74 years and older. Additionally, the effect of parental alcoholism among older individuals (eg, OR of 0.8 for participants aged 80 y) was significantly weaker than the effect among younger individuals (eg, OR of 2.3 for participants aged 60 y).

Age	Parental Alcoholism Lower Limit	Parental Alcoholism Upper Limit	Parental Alcoholism Estimate (Odds Ratio)
18.0	2.121333968	6.551176806	3.727899394
18.7272727	2.075574017	5.860781704	3.487762352
19.4545455	2.029848789	5.283819956	3.274958861
20.1818182	1.984035647	4.800238575	3.086072657
20.9090909	1.938049696	4.39400751	2.91818521
21.6363636	1.891863229	4.052213687	2.768796503
22.3636364	1.84552654	3.764360941	2.635759478
23.0909091	1.799186017	3.521828612	2.517225614
23.8181818	1.753093756	3.317455994	2.411599342
24.5454545	1.707603271	3.145230146	2.317499792
25.2727273	1.663149306	3.000060743	2.233729828
26.0	1.620212854	2.877621024	2.159249539
26.7272727	1.579278657	2.774236703	2.093153796
27.4545455	1.540794475	2.686804367	2.034653121
28.1818182	1.505138875	2.612721855	1.98305553
28.9090909	1.4726045	2.549829007	1.937753769
29.6363636	1.443395174	2.496351313	1.898215329
30.3636364	1.417631989	2.450842872	1.86397244
31.0909091	1.395365259	2.412132959	1.834613456
31.8181818	1.376588712	2.379277958	1.80977545
32.5454545	1.361253018	2.351518978	1.789137308
33.2727273	1.349275107	2.328241584	1.77241034
34.0	1.34054929	2.308950615	1.759335701
34.7272727	1.334956119	2.293250066	1.749682316
35.4545455	1.332367535	2.280824388	1.74324306
36.1818182	1.332650157	2.271423676	1.739831348
36.9090909	1.335667115	2.264852012	1.739278112
37.6363636	1.341277836	2.260956984	1.741428003
38.3636364	1.349333336	2.259616451	1.746131668
39.0909091	1.359678241	2.260738268	1.753247453
39.8181818	1.372151379	2.264258285	1.76264152
40.5454545	1.386584443	2.270136172	1.77418587
41.2727273	1.402800706	2.278351648	1.787756499
42.0	1.420613986	2.288900855	1.803231701
42.7272727	1.439825989	2.301790321	1.820488266
43.4545455	1.46021974	2.3170262	1.839393214
44.1818182	1.481565837	2.334616571	1.859808634
44.9090909	1.503626278	2.354567875	1.881592446
45.6363636	1.52615736	2.376878206	1.904597114
46.3636364	1.548913488	2.401529913	1.928668471
47.0909091	1.571651669	2.428481794	1.953644662
47.8181818	1.594132997	2.457658161	1.979351907
48.5454545	1.616121257	2.488935549	2.005597579
49.2727273	1.637394377	2.522142752	2.032176755
50.0	1.657749164	2.557057044	2.058873278
50.7272727	1.677002996	2.593401109	2.085459525
51.4545455	1.694993838	2.630842419	2.111696401
52.1818182	1.711578621	2.668995055	2.13733359
52.9090909	1.726627828	2.707421364	2.162107553
53.6363636	1.740019732	2.745634607	2.185739782
54.3636364	1.751641031	2.783109656	2.20794227
55.0909091	1.761383135	2.819292424	2.228419648
55.8181818	1.769137897	2.853610183	2.246871139
56.5454545	1.774793588	2.885483038	2.262992884
57.2727273	1.778231237	2.914336455	2.276480643
58.0	1.779321666	2.939615161	2.28703322
58.7272727	1.777923295	2.960796734	2.294356007
59.4545455	1.773879747	2.977405156	2.298164203
60.1818182	1.767017848	2.989025971	2.298186729
60.9090909	1.757146192	2.995321497	2.294170386
61.6363636	1.74405449	2.996045392	2.285884165
62.3636364	1.727514047	2.99105584	2.273123617
63.0909091	1.70727983	2.980326367	2.255715207
63.8181818	1.683095655	2.963954754	2.233521741
64.5454545	1.65470297	2.942168241	2.206448396
65.2727273	1.621849546	2.915316254	2.174443456
66.0	1.584304069	2.883859212	2.13750085
66.7272727	1.541875679	2.848350404	2.095662715
67.4545455	1.494437512	2.809410365	2.049021287
68.1818182	1.441952684	2.767695097	1.997720044
68.9090909	1.384500016	2.723860792	1.941953993
69.6363636	1.322295886	2.678528746	1.881969059
70.3636364	1.255708302	2.632254659	1.818060513
71.0909091	1.18525999	2.585505937	1.750570405
71.8181818	1.111619045	2.538649444	1.679884243
72.5454545	1.035577447	2.491949854	1.606426801
73.2727273	0.958018876	2.445576339	1.530656165
74.0	0.879880702	2.399616068	1.453057422
74.7272727	0.802113889	2.35409105	1.374135775
75.4545455	0.725644318	2.308975503	1.294409113
76.1818182	0.651338299	2.264211802	1.214400208
76.9090909	0.579974061	2.219723938	1.134628709
77.6363636	0.512220104	2.175428095	1.055603148
78.3636364	0.448620673	2.13124046	0.977813137
79.0909091	0.389588073	2.087082647	0.901721968
79.8181818	0.335401273	2.042885183	0.827759803
80.5454545	0.286210024	1.99858954	0.756317632
81.2727273	0.242043585	1.954149091	0.687742141
82.0	0.202823099	1.909529338	0.62233163
82.7272727	0.168376606	1.864707633	0.560333063
83.4545455	0.138455714	1.819672597	0.501940305
84.1818182	0.112752985	1.774423347	0.44729356
84.9090909	0.090919169	1.728968626	0.396479999
85.6363636	0.07257957	1.683325891	0.349535504
86.3636364	0.057348922	1.637520403	0.306447434
87.0909091	0.044844346	1.591584332	0.267158301
87.8181818	0.034696095	1.545555889	0.231570192
88.5454545	0.026555975	1.499478512	0.199549778
89.2727273	0.020103438	1.453400077	0.170933726
90.0	0.015049499	1.407372163	0.145534345

## Discussion

This study examined how the relationship between parental alcoholism and depression outcomes may change across individuals of different ages. Respondents who reported being exposed to parental alcoholism as children had approximately twice the risk of meeting criteria for lifetime MDD and PDD. Parental alcoholism had a positive and stable effect on the odds of lifetime MDD throughout most of the age range of the participants, although this association was no longer significant for those aged 85 years old or older. However, although the association with PDD was positive and stable across individuals in early and late adulthood, it significantly decreased in strength for those older than 73, such that parental alcoholism was no longer associated with a heightened risk for PDD.

Results of this study also showed that 23% of adults had a parent with alcohol problems before the age of 18; the 1988 National Health Interview Survey estimated that 18.1% of adults had a parent with alcohol problems before the age of 18 (10). Although there is a large gap in timeline, the prevalence of adults growing up with a parent with alcohol problems seems comparable. Although current data on the prevalence of adults who grew up with a parent with alcohol problems are not available, it is estimated that an annual average of 7.5 million US children (10.5% of all children) live with a parent who had an alcohol use disorder in the past year (11). Although this figure is lower than we report here, it includes only past-year alcohol use disorder, a severe form of problem drinking. Hence, assuming that this prevalence will increase under NESARC’s inclusion of other, less severe forms of problem drinking, the current prevalence rates are more consistent with those of previous reports.

Our findings confirm those of previous research that established that parental alcoholism is associated with an increased risk of depression among offspring (2,12,13). This study also extends this research in 2 important ways, given that many previous studies are limited to younger adults (2,3). Here, we examined the effects of parental alcoholism on depression among adults across a wide age range, and we rigorously examined the age-varying effects of parental alcoholism, showing that its effect is largely stable across individuals from early to late adulthood.

This study has limitations. First, the measure of parental alcoholism is limited in several ways. The single question that assessed parental alcoholism was proxy-reported by offspring. As a result, both the timing and the nature of the question may have created recall bias, in which those with depression are more likely to remember the drinking of their parents as problematic than those with no depression. Additionally, the wording of the question included parents as well as non-parental adults living in the household, although most participants reported living only with one or more biological parents. Thus, the wording of this question may have affected the results in unknown ways. Second, this study used cross-sectional data and thus cannot conclude that parental alcoholism causes depression among offspring.

Third, because we used cross-sectional data, the findings do not distinguish between true age and cohort when considering the age-varying effect of parental alcoholism. A true age-varying effect would capture data on the change in the effect of parental alcoholism as an individual ages, but these analyses examined the effect across individuals of different ages. This analysis introduces a cohort effect: the association between parental alcoholism and depression may change across individuals born in different years as a result of differences across time periods in, for example, the prevalence of parental alcoholism, the threshold at which participants consider alcohol consumption “problem drinking,” the prevalence of depression, or other associated risk and protective factors. It is likely that both an age effect (5) and a cohort effect (14,15) contribute to our findings, but this study cannot distinguish between them. Thus, the findings should not be interpreted as effects for a given individual across time. Future studies using longitudinal data are needed to separate true age-varying effects from cohort effects.

Strengths of this study include the large, nationally representative sample, the use of rigorous and well-validated DSM-5 measures of MDD and PDD, and the use of TVEMs, an innovative methodology for examining continuous moderation across age.

Parental alcoholism is stably associated with depression outcomes among offspring across a range of ages from early to late adulthood, with a decline in PDD among older adults. This finding implies that the effect of parental alcoholism on PDD may weaken among older adults (aged ≥60 y), making them more resilient than middle-aged and younger adults for PDD. Conversely, we found no evidence of resilience to MDD, as shown by a similar effect across ages. Despite this long-term effect of parental alcoholism, many adults with depression do not seek treatment because of a desire for self-reliance and the perceived stigma of mental health difficulties (16). Children of alcoholics often desire secrecy about their parents’ alcoholism (17), and this additional stigma may further compound the lack of treatment seeking among adult offspring of alcoholics. Our findings highlight the importance of screening for depression among offspring of alcoholics in health care settings to provide them with services and support to ultimately manage this mental health burden.

## Post-Test Information

To obtain credit, you should first read the journal article. After reading the article, you should be able to answer the following, related, multiple-choice questions. To complete the questions (with a minimum 75% passing score) and earn continuing medical education (CME) credit, please go to http://www.medscape.org/journal/pcd. Credit cannot be obtained for tests completed on paper, although you may use the worksheet below to keep a record of your answers.

You must be a registered user on http://www.medscape.org. If you are not registered on http://www.medscape.org, please click on the “Register” link on the right hand side of the website.

Only one answer is correct for each question. Once you successfully answer all post-test questions, you will be able to view and/or print your certificate. For questions regarding this activity, contact the accredited provider, CME@medscape.net. For technical assistance, contact CME@medscape.net. American Medical Association’s Physician’s Recognition Award (AMA PRA) credits are accepted in the US as evidence of participation in CME activities. For further information on this award, please go to https://www.ama-assn.org. The AMA has determined that physicians not licensed in the US who participate in this CME activity are eligible for *AMA PRA Category 1 Credits™*. Through agreements that the AMA has made with agencies in some countries, AMA PRA credit may be acceptable as evidence of participation in CME activities. If you are not licensed in the US, please complete the questions online, print the AMA PRA CME credit certificate, and present it to your national medical association for review.

## Post-Test Questions

### Study Title: Time-Varying Effects of Parental Alcoholism on Depression

#### CME Questions

1. Your patient is a 37-year-old man with a history of maternal alcoholism. On the basis of the national database study using the National Epidemiological Survey on Alcohol and Related Conditions, wave III, by Thapa and colleagues, which one of the following statements about risk for lifetime major depressive disorder (MDD) among children of alcoholic parents is correct?
  - Parental alcoholism was associated with nearly twice the risk for MDD (OR, 1.98; 95% CI, 1.85-2.11; *P *< .001)
  - The association between parental alcoholism and MDD declined with increasing age above 50 years
  - *Diagnostic and Statistical Manual of Mental Disorders, 5th edition* (DSM-5), criteria for MDD were met by 15% of those who reported parental alcoholism and by 6% of those who did not
  - Age at first episode of MDD was not significantly different between those who reported parental alcoholism and those who did not
2. According to the national database study using the National Epidemiological Survey on Alcohol and Related Conditions, wave III, by Thapa and colleagues, which one of the following statements about risk for lifetime persistent depressive disorder (PDD) among children of alcoholic parents is correct?
  - Parental alcoholism was not associated with a significantly higher risk for PDD
  - The association with PDD significantly declined among older adults, with those older than 73 years no longer at increased risk for PDD
  - DSM-5 criteria for PDD were met by 12% of those who reported parental alcoholism and by 3% of those who did not
  - Age at first episode of PDD was not significantly different between those who reported parental alcoholism and those who did not
3. According to the national database study using the National Epidemiological Survey on Alcohol and Related Conditions, wave III, by Thapa and colleagues, which one of the following statements about clinical implications regarding risk for lifetime depression among children of alcoholic parents would be correct?
  - The study proves that parental alcoholism causes depression among offspring
  - Given the low prevalence of parental alcoholism, it is unlikely to cause a significant public health burden in the offspring
  - Adult offspring of alcoholics are more likely to seek treatment for depression than those without this family history
  - The findings support the need to screen adults with parental alcoholism for depression to provide them with services and support to manage this mental health burden
